# Empirical determination of the effective solid modulus in organic-rich shales

**DOI:** 10.1038/s41598-023-45393-9

**Published:** 2023-10-26

**Authors:** K. Larkin Spires, John P. Castagna, Sheyore John Omovie

**Affiliations:** University of Houston, Houston, TX 77004 USA

**Keywords:** Geology, Geophysics

## Abstract

Calculating the change in the saturated bulk modulus of a saturated rock with new fluid properties requires a priori selection of an effective bulk modulus of the solid constituents. When the rock constituents have similar mineral moduli, the theoretical bounds on the solid modulus are close to each other. However, when solid properties vary greatly, as in organic-rich shales, the actual effective solid modulus of a physical rock may vary significantly between the bounds which results in uncertainty in the predicted change in the saturated bulk modulus of the rock. We use a semi-empirical rock physics model utilizing the Brown–Korringa equation for mineralogically heterogenous rocks and introduce three parameters to estimate the pore space compressibility, the dry frame compressibility, and the fractional position of the effective solid modulus relative to the Reuss and Voigt bounds. We optimize for these three parameters in seven organic shale formations and find that the Reuss bound for the effective solid material modulus best fits the data when organic content is high. Furthermore, we use this model to fluid substitute to 100% brine saturation and find Gassmann’s equation using the Hill average predicts similar saturated moduli to the semi-empirical Brown–Korringa rock physics model when volume fraction of solid organic matter is less than 5%. However, at higher organic contents, we find that the error using the Gassmann–Hill approach increases, and the semi-empirical Brown–Korringa model better fits the data.

## Introduction

Fluid substitution using Gassmann’s equations^1,2^ is a frequently used procedure in the petroleum industry for applications such as direct hydrocarbon detection (e.g.^3^) through forward modeling, carbon sequestration (e.g.^4^) in determining the brittleness of seals and modeling CO2 plume migration in storage sites, source rock evaluation (e.g.^5^), and elastic moduli and brittleness estimation in unconventional shale reservoirs (^6,7^). Gassmann^1^ expresses the bulk modulus of a fluid saturated rock as a function of the porosity, the bulk modulus of a single solid constituent, the pore fluid modulus, and the bulk modulus of the rock frame (skeleton). Gassmann^1^ assumes a homogenous solid and does not address what effective solid material bulk modulus to use when the composite is an aggregate of minerals with different moduli. In practice (e.g.^3,8,9^) the Reuss and Voigt bounds of the constituent moduli are averaged to form the Reuss–Voigt–Hill effective solid material modulus^10^. The wider the range of constituent moduli, the more opportunity for non-linearity and use of the Hill^10^ average to introduce error into Gassmann fluid substitution. In organic shales containing highly compressible kerogen there can be an order of magnitude variation in the moduli of solid materials, so the question of how to properly average constituent moduli can be significant.

Berryman and Milton^2^ deal with two mineral constituents by relating the modulus of the porous composite frame to the moduli of the minerals and the porous monomineralic frames of each of the two minerals. Unfortunately, the individual mineral porous frame properties are generally not known for practical applications involving in situ rocks. Furthermore, for more than two minerals, the mathematics becomes unwieldy. Despite this lack of an explicit theoretical formulation for the effective solid, there is still the need to perform fluid substitution in situ for rocks encountered in a borehole containing both hard and soft solid constituents..

Thomsen^11^ points out an error in Gassmann’s derivation of the fluid substitution equation and indicates that the Brown and Korringa^12^ formulation is correct. Furthermore, the Brown and Korringa equation allows for compositional heterogeneity in the solid constituents, while there is no theoretical basis for doing so via Gassmann’s equation. Unfortunately, Brown and Korringa introduce two elastic constants that are conceptually poorly understood and often not readily available for practical applications (see Eq. (2), below) which adds an additional unknown modulus to the fluid substitution problem. Our literature search only produced examples applying Brown and Korringa to laboratory samples or numerical models.

The purpose of this paper is to apply Brown and Korringa fluid substitution to subsurface data without laboratory calibration for the missing Brown and Korringa moduli. In the absence of explicit theoretical guidance, we gave particular emphasis on the effective solid material compressibility to use for fluid substitution in organic shales having a wide range of solid constituent moduli. We do so by optimizing coefficients to empirical equations for seven different unconventional reservoirs with varying composition and hydrocarbon content including organic shales from the Wolfcamp, Cline, Bakken, Eagle Ford, Woodford, Avalon, and Spraberry formations^7,9,13^, and finding empirical moduli relationships that best fit all the data in each formation.

Muller and Sahay^14^ introduce an additional unknown in the fluid substitution process, which is the coefficient, *n*. In this paper, we assume that *n* = 1 to limit the number of degrees of freedom in fitting the data and to reduce the fluid substitution problem exactly to the Brown and Korringa formulation. We will treat variation of this parameter, if any, in future research. Furthermore, as a first step, we deal only with velocities measured normal to bedding. Further work would then be required to apply our results to velocities measured with other orientations.

## Theory

### Bounding equations and the Hill average for effective solid moduli

**Figure 1:**
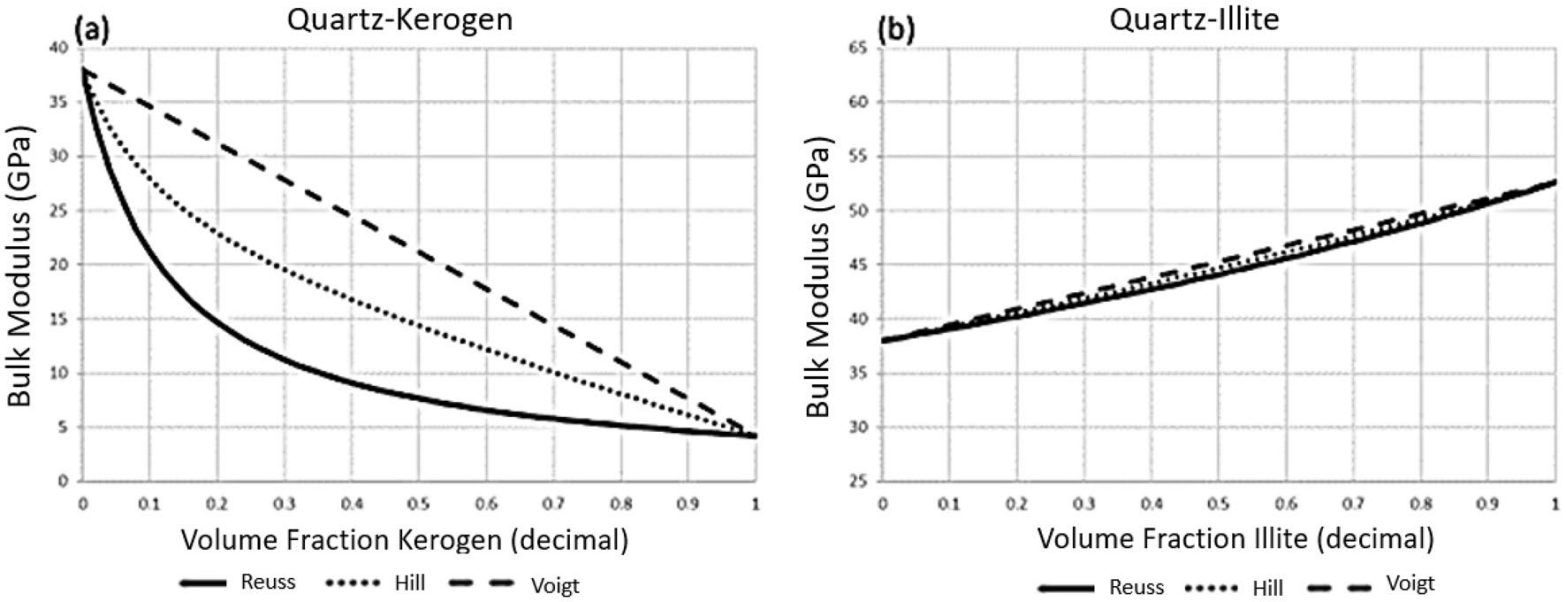
Examples of Reuss–Voigt bounds and the Hill average in zero-porosity mineral composites (**a**) quartz–kerogen mixture with almost an order of magnitude difference in bulk moduli between bounds, and (**b**) quartz–illite mixture with tight bounds. The closer the constituent moduli, the tighter the bounds and the better the Hill average is as an estimate of the modulus of the composite.

The widest possible bounds for a zero-porosity isotropic polycrystalline assemblage of randomly oriented and distributed isotropic constituents are given by the Reuss (lower bound) and Voigt (upper bound) averages corresponding to volume weighted reciprocal (Reuss) or linear (Voigt) averages of the constituent moduli (see^8^). In the special case of equal constituent shear moduli, the Hill^10^ arithmetic average of the Reuss and Voigt bounds is exact, as the bounds coincide in that specific case. Figure 1 shows the Reuss and Voigt bounds of the mineral modulus of two numerical rocks with zero porosity along with the calculated Hill average. The vertical scales of the graphs are the same which clearly shows the difference between the Reuss and Voigt bounds when varying from 100% quartz to 100% kerogen (Fig. 1a) and to 100% illite (Fig. 1b). When constituent bulk moduli are similar as in Fig. 1b, the bounds are narrow, and the Hill average is a good approximation; even when shear moduli are unequal. However, when constituent moduli differ by an order of magnitude as in Fig. 1a, the bounds are wide, and the Hill average is likely to be incorrect by a significant amount; in the vicinity of ± 8 GPa in a binary mixture of about equal amounts of quartz and kerogen (see Fig. 1a). According to Mavko and Mukerji^15^, use of the Hill average is only appropriate in Gassmann fluid substitution when the ratio of the bulk moduli of the softest components to the stiffest is greater than 0.3.

It is possible to invoke tighter theoretical bounds^16^. However, these Hashin–Shtrikman bounds for solid bulk modulus require knowledge of both bulk and shear moduli of all solid constituents. In application to organic shales, this requires knowledge of the shear modulus of kerogen, which is highly variable with depth and degree of maturity, and usually poorly known. It is not clear that the Hashin–Shtrikman bounds would be applicable in an anisotropic finely laminated medium, as shales can be. Furthermore, it is possible for the effective mineral modulus in the Brown-Korringa equation to violate bounding equations if not all solid constituents are “participating”^12^ in structural support of the rock frame. Rather than invoking a particular averaging scheme, in this paper we will optimize a rock physics model, based on the Brown and Korringa^12^ fluid substitution formulation (which is applicable for composites with multiple solid constituents), for the local Brown and Korringa moduli relationships that best fit the observations in various organic shale formations.

In addition to the case of constant shear modulus, there are two relevant situations where the exact theoretical effective zero-porosity solid modulus is known:

1. As the shear modulus of an interpenetrating medium surrounding the other constituents goes to zero, the effective modulus of the resulting “suspension-like material” approaches the Reuss bound^17^. This could be relevant where total organic content is high and continuously distributed throughout the composite, as kerogen typically has a relatively low shear modulus on the order of 3 GPa^18^ (compare with the other minerals in Tables 2 and 3).
2. If the constituents are individually arranged to form a series of very thin single constituent isotropic layers (each of which is a small fraction of a wavelength in thickness) and wave propagation is normal to the bedding, this also corresponds to the Reuss bound^8^. Both situations may be approached to some extent in shales with high organic content.

In this paper, we use velocities acquired normal to bedding and ignore anisotropy in the fluid substitution process. This procedure, although common in the literature, is open to criticism, c.f. Thomsen^11^. We use it here nonetheless since the logs do not provide enough information to describe the anisotropy adequately. Given sufficient information, further improvement in our model may be possible using the Brown and Korringa fully anisotropic fluid substitution equations.

### Fluid substitution

Gassmann^1^ considers the mechanical dependence of the static bulk modulus of a fluid-saturated porous isotropic-composite (${K}_{ud}$) on the bulk modulus of the fluid (${K}_{F}$) the bulk modulus of the solid mineral (${K}_{S}$) the bulk modulus of the “dry” frame (${K}_{fr}$), and the porosity ($\phi$) (e.g.,^8^):

1 $$\frac{{K}_{ud}}{{K}_{S}-{K}_{ud}}=\frac{{K}_{fr}}{{K}_{S}-{K}_{fr}}+\frac{{K}_{F}}{{\phi (K}_{S}-{K}_{F})}$$

Another outcome of Gassmann’s theory is that the shear modulus is independent of the fluid modulus^2^.

The use of the adjective “dry” to describe the frame modulus is somewhat of a misnomer, as it is the drained frame modulus in the presence of the wetting fluid, and thus the term “dry” ignores physico-chemical solid–fluid interactions that may cause the modulus to change with saturating fluid. Furthermore, the act of drying a rock can change the frame properties. We use the term “frame” modulus to be consistent with Thomsen’s publication^11^. Biot^19^ used the term “skeleton” to describe this modulus. In our application, the frame modulus is that which is in contact with the wetting fluid, which we assume will remain so as fluid saturation changes.

Equation (1) ignores dispersion and is strictly valid only at zero frequency. However, Omovie and Castagna^13,20^ argue and present empirical evidence that Gassmann’s equations are not violated by observations in low permeability shales and that no statistically significant dispersion between their core and well log measurements is observed. Gassmann assumes fluid pressure equilibration between the pores, so the porosity can be taken to be the acoustically connected porosity, with any disconnected porosity assigned to the solid matrix^12,21^. Fluid pressure equilibration can be achieved, even in disconnected pores, if pores under compression strain to the same degree. For example, perfectly spherical disconnected pores under compression will obey Gassmann’s equation in a monomineralic rock^22,23^. Similarly, if the medium has aligned flat pores parallel to bedding, as might be expected to a large extent in a shale for wave propagation perpendicular to bedding, one would expect similar strain in the aligned “cracks”. Omovie and Castagna^13,20^ also reason that, according to the general Biot^19^ theory, acoustic measurements fall well below the critical frequency calculated for shales and that dispersion can be safely ignored. Thus, it is feasible that Eq. (1) is applicable to shales in particular circumstances.

In performing Gassmann’s fluid substitution in practice, the measured or otherwise known in situ variables *K*_*ud*_, *K*_*F*_, *K*_*S*_, and *f* are used to solve for *K*_*fr*_, then the derived *K*_*fr*_, new *K*_*F*_, and original *K*_*S*_ and *f* are used to calculate the predicted *K*_*ud*_ with the new fluid. Smith et al.^3^ provides a detailed tutorial of the fluid substitution process.

As the mineral modulus is unknown when there are multiple solid constituents, it is common practice to use the Hill^10^ average of constituent moduli (e.g.^3,8,15,24^) as the effective solid constituent modulus. We refer to this approach as Gassmann–Hill fluid substitution.

Alternatively, the Brown and Korringa^12^ approach assumes an arbitrary mixture of mineral constituents and is written in terms of the compressibilities. Specifically,

2 $$\frac{1}{{C}_{ud}-{C}_{M}}=\frac{1}{{C}_{fr}-{C}_{M}}+\frac{1}{{\phi \left(C\right.}_{F}-{C}_{\phi })}$$

in which ${{c}_{\phi }}$ is the compressibility of the pore space with constant differential pressure as fluid pressure varies, ${c}_{fr}$ is the compressibility of the dry rock frame, equal to 1/ *K*_*fr*_, the mean compressibility ${c}_{M}=\left(1-\phi \right){c}_{S}+\phi {c}_{\phi }$, where ${c}_{S}$ is the effective compressibility of the mixture of solid material comprising the solid rock matrix,${c}_{ud}$ is the compressibility of the undrained saturated rock, and ${c}_{F}$ is the compressibility of the fluid. There is no general theoretical expression to calculate ${c}_{S}$ from the solid constituent moduli, as it will depend on the constituent micro-geometry as well as the contrast in moduli^15,25^. In practice, the Hill^10^ Voigt–Reuss–Hill average is used for the mineral bulk modulus with little error when constituents have similar moduli^15^. However, in organic shales with an order of magnitude range of mineral moduli, and absent detailed microstructural information or laboratory calibration to associate with logging measurements, and with insufficient theoretical constraint, we must resort to empirical methods.

### Empirical rock physics model

Given the porosity and the compressibility of the saturated composite (${C}_{\rm{ud}}$) and pore fluid (${C}_{\rm{F}}$), there are three unknowns in Eq. (2): ${C}_{S}$, ${C}_{\upphi }$, and ${C}_{fr}$. Assuming the effective solid material bulk modulus, ${K}_{S}$ = *1/*${c}_{S}$, must lie between the Reuss (${K}_{Reuss}$) and Voigt (${K}_{Voigt}$) bounds, we introduce an arbitrary empirical parameter ξ, which specifies any allowable value of ${K}_{S}$ as a weighted average of the bounds:

3 $${K}_{S}= \xi {K}_{Reuss}+\left(1-\xi \right){K}_{Voigt}$$

When $\xi =1$, the effective solid modulus is equal to the Reuss bound. Similarly, when $\xi =0$ the effective solid modulus is equal to the Voigt bound, and when $\xi =0.5$ it is the Hill average. As $\xi$ is a continuous variable that can take on any value, it is readily optimized to empirically fit data. In the data presented here, we did not find optimized values outside the range 0 to 1 although that is theoretically possible^12^.

Following Kachanov et al.^26^, the pore space compressibility in a monomineralic solid is given by

4 $${{c}_{\phi }=pc}_{S}$$

where $p$ is the “pore shape factor”, which we extend here to a heterogenous material and treat as a strictly empirical coefficient, again optimized by fitting the data. Vernik^27^ describes the theoretical relationship between *p* and pore shape which will be discussed further below. In the Brown and Korringa formulation^12^, the pore space compressibility is at constant differential pressure as pore pressure is varied as opposed to the dry rock pore compressibility. We must emphasize, therefore, that in our application of Eq. (4) we view *p* only as an empirical coefficient that may possibly be only loosely related to pore shape at the average composition of the rocks for which it is optimized.

We add an additional degree of freedom to relate the dry frame compressibility to the effective solid compressibility by using the Nafe and Drake^28^ empirical relation:

5 $${c}_{fr}=\frac{{C}_{S}}{{\left(1-\phi \right)}^{m}}$$

where $m$ is a locality dependent empirical coefficient. Presumably, at a given locality and associated pressures, the exponent *m* could vary with degree of lithification/cementation, pore shape, and composition (especially clay content and total organic carbon).

Equations (2)–(5) define a rock physics model, with empirical coefficients $\xi$, $p$, and $m$, that can be obtained by fitting well log compressional and shear-wave velocities and density in any specific locality, formation, and depth interval.

### Data

The data used in this study come from vertical wells drilled in seven unconventional organic shale formations in the continental United States: the Spraberry, Avalon, Cline, Eagle Ford, Wolfcamp, Woodford and Bakken formations. Each well has a full well logging suite and volumetric analysis including total organic content and fluid saturation calibrated with core measurements along with reliable compressional and shear wave logs. These data were previously reported and fully described in Vernik et al.^9^ and Omovie and Castagna^7,13^, including the logs as supplementary online data) and summarized in Table 1. Solid constituent volume fractions reported include kerogen, pyrite, calcite, dolomite, clay, and quartz. We loosely include all the solid organic matter in the kerogen volume fraction and feldspars are included in the quartz fraction. Mean velocities and derived moduli are only provided in Table 1 to describe where the data are clustered and are not meant to have strict physical significance. Tables 2 and 3 shows the solid and fluid properties used by formation.

**Table 1:** Average rock properties from well logs for shale reservoirs studied here.

	Depth (m)	Density (gm/cc)	Vp (km/s)	Vs (km/s)	Vp/Vs (ratio)	mu (GPa)	K (GPa)	TOC (wt%) (percent)	Depth range (m)
Formation									
Spraberry	2007	2.56	3.74	2.17	1.72	12.04	19.68	2.88	1962–2046
Wolfcamp	2350	2.54	3.73	2.16	1.73	11.84	19.74	3.71	2326–2374
Avalon	2380	2.46	3.77	2.34	1.61	13.60	17.24	7.61	2337–2423
Woodford	3193	2.52	3.51	2.11	1.66	11.25	16.02	5.58	3188–3200
Eagle Ford	3886	2.55	4.06	2.39	1.70	14.60	22.67	2.88	3838–3928
Cline	2807	2.52	3.70	2.24	1.66	12.64	17.82	6.07	2772–2831
Bakken	3381	2.28	3.05	1.84	1.66	7.75	11.01	14.44	3371–3391

	XTOC (decimal)	XPyrite (decimal)	XCalcite (decimal)	XDolomite (decimal)	XClay (decimal)	XQuartz (decimal)	ϕ_Total_ (decimal)	Sw (decimal)	Ro (percent)
Spraberry	0.05	0.03	0.09	0.00	0.33	0.49	0.10	0.54	0.80
Wolfcamp	0.07	0.03	0.11	0.00	0.27	0.51	0.08	0.44	0.80
Avalon	0.14	0.03	0.02	0.02	0.20	0.60	0.07	0.30	0.94
Woodford	0.11	0.02	0.00	0.00	0.35	0.53	0.04	0.47	0.98
Eagle Ford	0.06	0.01	0.60	0.00	0.10	0.15	0.08	0.49	1.80
Cline	0.12	0.03	0.03	0.01	0.32	0.48	0.06	0.55	0.98
Bakken	0.26	0.03	0.00	0.12	0.21	0.38	0.07	0.24	1.30

Each formation is represented by a single well, so these values are not meant to be representative of the entire geographic extent of the formations. High water saturations (greater than 70%) and low TOC volume fractions less than 1% are excluded. Reported quantities are mean values for included depths. Depth is mean depth for included datapoints in each formation. Density is mean logged density. Vp and Vs are mean sonic compressional and shear-wave velocities. μ and k are derived shear and bulk moduli from mean velocities and density. Ro is percent vitrinite reflectance determined from cuttings and S_w_ is mean decimal water saturation from log analysis. The X_i_ are mineral solid volume fractions from volumetric log analysis such that ∑X_i_ = 1 for organic matter (XTOC), pyrite (X_Pyrite_), calcite (X_Calcite_), dolomite (X_Dolomite_), clay (X_Clay_) and quartz plus feldspars (X_Quartz_). TOC is weight percent of total solid organic carbon. XTOC is the volume fraction of solid organic carbon derived from TOC using the Vernik^27^ relation. The depth range is the top and base of the logged interval. ϕ_Total_ is total porosity using measured log density, fluid density, and grain density from solid volume fractions^7^.

**Table 2:** Average fluid properties by formation.

Formation	Hydrocarbon type	${K}_{HC}$ (GPa)	${\rho }_{HC}$ (g/cc)	${K}_{Brine} ($GPa)	${\rho }_{Brine} ($g/cc)	Mean temperature (°C)	Pore pressure (MPa)
Avalon	Volatile oil	0.366	0.670	2.75	1.05	50	30
Bakken	Oil	0.750	0.800	2.75	1.05	80	46
Cline	Volatile oil	0.430	0.675	2.80	1.05	54	35
Eagle Ford	Gas	0.130	0.250	2.80	1.05	93	53
Spraberry	Volatile oil	0.375	0.675	2.75	1.05	45	21
Wolfcamp	Volatile oil	0.434	0.677	2.73	1.05	49	28
Woodford	Volatile oil	0.430	0.675	2.75	1.05	68	36

Fluid properties are calculated from the Batzle and Wang^29^ equations for the formation fluid composition and in situ pore pressure and temperature.

**Table 3:** Mineral end member elastic moduli used for computing matrix bulk modulus for fluid substitution.

Mineral	${\rho }$ (g/mL)	${K}$ (GPa)	${\mu }$ (GPa)
Kerogen	1.300	5.53	3.20
Pyrite	4.930	147.63	129.04
Calcite	2.712	64.51	27.72
Dolomite	2.870	91.76	35.92
Clay	2.780	52.60	31.50
Quartz	2.649	38.00	41.77

Kerogen is from Vernik and Landis^27^ and used to represent all solid organic matter.

## Method

As shales are highly anisotropic for orientations that are not normal to bedding, the anisotropic Brown and Korringa^12^ fluid substitution equations should be applied, and recalibration of empirical coefficients should be made according to orientation. In this study, as a first approximation and to keep the number of data fitting degrees of freedom small, we deal with only vertical velocities obtained in straight boreholes with only minor formation dip (bedding normal). To verify and simplify the fluid substitution approach, we consider only the vertical component with the understanding that extension to consider anisotropic effects is feasible with additional constraint or optimized parameters. The “isotropic parameters” thus constructed should be regarded as “effective” isotropic parameters.

In our approach to fluid substitution, the well log acoustic compressional-wave and shear-wave velocities and density are combined to extract the dynamic saturated modulus, *K*_*ud*_, at each well log depth. Core-calibrated well log volumetric analyses reported by Omovie and Castagna (^7^ with data provided online) provide porosity, solid constituent volume fractions, and fluid saturations. The effective solid Reuss and Voigt moduli, ${K}_{Reuss}$ and ${K}_{Voigt}$, are calculated given the constituent volume fractions and material properties in Table 1. Fluid moduli used are based on fluid composition and in situ conditions using the Batzle and Wang^29^ equations. The effective fluid modulus, *K*_*F*_, at the well log water saturation, is calculated using the Reuss bound^17^ with locality dependent fluid moduli given in Table 2.

Over a given well log depth interval in an organic shale, if empirical coefficients ξ, *p*, and *m* are assumed to be constant over the interval, then with potentially hundreds of well log observations the three unknown constant coefficients are readily obtained by exhaustive search. If the observed *K*_*ud*_ values are reproduced with low mean-squared error and pass statistical significance tests, then the optimized coefficients can be loosely interpreted as weighted average values for the interval of interest.

For each of the seven organic shale formations comprising the data used in this study, the three empirical coefficients were optimized by allowing them to vary over a specified range and minimizing the mean square error between the fitted *K*_*ud*_ and the measured *K*_*ud*_. *m* was varied over a range from 1 to 20 in increments of 0.25, $p$ was varied from 1 to 40 in increments of 0.5, while $\xi$ was varied from 0 to 1 in increments of 0.05. For each trial combination of $\xi$, $p$, and $m$, the Brown and Korringa^12^ compressibilities were calculated from Eqs. (3)–(5) and inserted into Eq. (2). The combination of $\xi$, $p$, and $m$ that exhibited the minimum mean squared error between predicted and measured saturated moduli at in situ saturation was selected as the best combination for each formation. As the trials include all possible combinations of the coefficients, the result is a global minimum without any subjective interpretive bias guiding convergence towards a preferred outcome. The best combination was then used to perform fluid substitution for all depths in the interval via Eq. (2).

## Results

**Figure 2:**
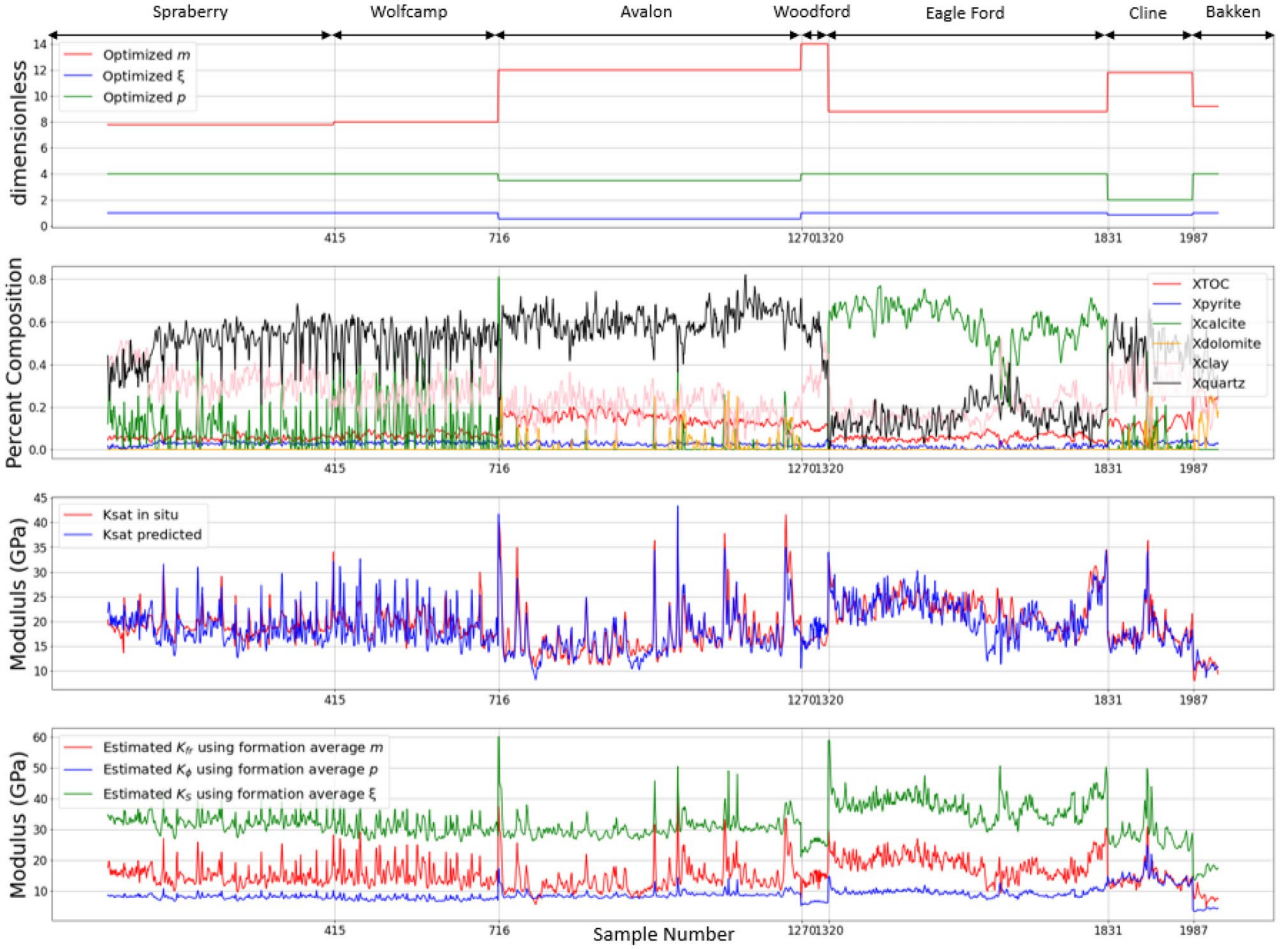
Concatenated optimization results plotted as logs for the seven formations. The horizontal axis is the sample number which is a proxy for depth. Measured and optimized well logs are sampled every half foot (0.1524 m) over the depth range given for each formation in Table 1. The formation names and ranges are given above the top panel. Sample numbers are 1–414 for the Spraberry, 415–715 for the Wolfcamp, 716–1269 for the Avalon, 1270–1319 for the Woodford, 1320–1830 for the Eagle Ford, 1831–1986 for the Cline, and above 1987 for the Bakken. Top panel: optimized empirical coefficients *m*, *ξ*, and *p* for each formation. Second panel: the X_i_ are mineral solid volume fractions from volumetric log analysis such that ∑X_i_ = 1 for organic matter (XTOC), pyrite (X_Pyrite_), calcite (X_Calcite_), dolomite (X_Dolomite_), clay (X_Clay_) and quartz plus feldspars (X_Quartz_). TOC is weight percent of total solid organic carbon. XTOC is the volume fraction of solid organic carbon derived from TOC using the Vernik^27^ relation. Third panel: red curve—dynamically measured *K*_*ud*_ from sonic and density logs. Blue curve—predicted *K*_*ud*_ using the semi-empirical Brown–Korringa model (Eqs. (2)–(5)), volumetric well log analysis, and the optimized coefficients for each formation. Bottom panel: predicted Brown–Korringa bulk moduli using the optimized coefficients in each formation. Red curve—dry frame modulus, *K*_*fr*_. Blue curve—pore space modulus, *K*_φ_. Green curve—effective solid constituent bulk modulus, *K*_*s*_.

**Figure 3:**
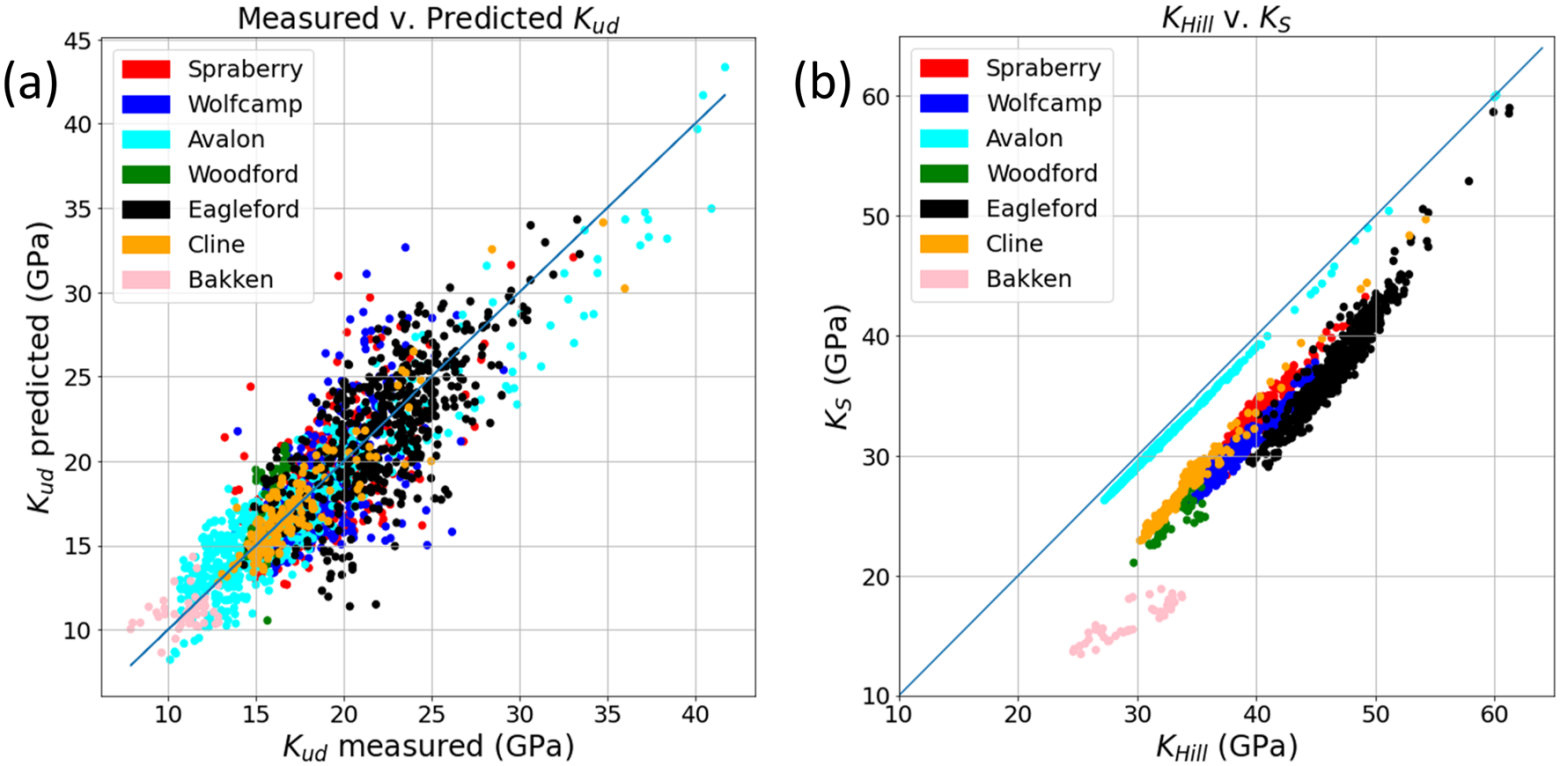
(**a**) Dynamically measured *K*_*ud*_ from sonic and density logs compared to predicted *K*_*ud*_ using the semi-empirical Brown–Korringa model (Eqs. (2)–(5)), volumetric well log analysis, and the optimized coefficients for each formation colored by formation. (**b**) Effective mineral modulus, *K*_*s*_, obtained from the optimized ξ for each formation versus the Hill average, *K*_*Hill*_. The Hill average is always greater than *K*_*s*_ in these organic shales.

The optimization by formation for $\xi$, $p$, and $m$ is shown graphically in Figs. 2 and 3 as well as numerically in Table 4. In Fig. 2, the top panel shows the values derived per formation, the second panel shows mineralogic composition including kerogen, the third shows the in situ undrained bulk modulus calculated from sonic and density logs alongside the predicted value from the optimization, the fourth panel displays the calculated missing moduli from the parameters. Figure 3a the in situ undrained bulk modulus against the predicted. Figure 3b plots the hill average against the optimized effected mineral modulus showing that the Hill average is an over-estimate. Table 4 contains the optimized values of $\xi$, $p$, and $m$ broken down by formation along with the statistical measures of significance including RMSE, R, F-statistic, and p-value. Should the least mean-squared error minimum not be well defined, we would expect the inverted parameters to vary in an unstable manner between formations. To the contrary, the coefficients vary in a reasonable way given the variable geological conditions. The predicted saturated bulk modulus using only the three free parameters ($\xi$, $p$, and $m$) for each formation is excellent (a root-mean-squared error of 2.59 GPa with a correlation coefficient of 0.84 for the entire dataset) and statistically significant relative to an alpha significance level of 0.05 (the F-statistic is 231 with a near zero p-value) It should be noted that the sampled data and predictions are non-Gaussian (multi-modal and skewed). This is expected of the data populations that span a variety of lithologies in each interval. Our statistical tests are thus not strictly accurate and should be viewed as indicators of significance only. The match observed in the third panel of Fig. 2 and crossplot in Fig. 3a is impressive given the limited number of free parameters and the fact that part of the mismatch can be attributed to experimental error in the velocities and densities used to extract the observed *K*_*ud*_ and in the volumetric compositional analysis used as input to the rock physics model. As indicated in Table 4, when the coefficient optimization is performed with only three constant coefficients for the entire dataset, the goodness of fit is slightly reduced, but the F statistic increases to 1260 as all 2033 datapoints are fit with only three free parameters.

**Table 4:** Empirical coefficients optimized by formation and resulting indicators of goodness of fit to the measured dynamic bulk modulus, *K*_*ud*_.

Samples	Formation	${\xi }$	*m*	*p*	RMSE	R	F statistic	p*-*value
554	Avalon	0.55	12.00	3.5	1.92	0.93	1263	0
47	Bakken	1.00	9.25	4.0	1.42	0.31	1.56	0.21
156	Cline	0.85	11.75	2.0	1.35	0.91	239	0
511	Eagle Ford	1.00	8.75	4.0	2.76	0.69	158	0
414	Spraberry	1.00	7.75	4.0	2.42	0.56	63.0	0
301	Wolfcamp	1.00	8.00	4.0	2.96	0.54	40.0	0
50	Woodford	1.00	14.00	4.0	2.74	0.49	4.74	0.0055
2033	All—average over all formations	N/A	N/A	N/A	2.59	0.84	231	0
2033	All—optimized as a single set	1.00	8.00	3.5	2.94	0.80	1260	0

RMSE is root-mean-squared error in GPa, R is the correlation coefficient, F is the Fisher F-statistic, the p-value is the single tailed probability that a model with no independent variables fits the data as well as the applied rock physics model. The only statistical concern is the Bakken, where the correlation coefficient is low because the range of moduli is small and the prediction fails an alpha confidence level of 0.05, although the fit to the data is good and F exceeds unity. Combining the results for all the formations with the coefficients determined by formation (a total of 21 coefficients with 3 in each formation) yields good statistics. Optimizing for only 3 coefficients for all formations combined has slightly reduced goodness of fit but greatly increased F due to the large number of datapoints with very few degrees of freedom.

It is important to note that the best fit $\xi$ parameter is at or close to unity in six of the seven formations—this suggests that, in contrast to standard fluid substitution practice using the Hill average, in organic shales the Reuss average of the constituent mineral moduli is usually a better choice than the Hill average in Brown–Korringa fluid substitution. Similarly, Wang et al.^30^ utilize an inclusion-based rock physics model and account for organic matter using the Backus average, which is virtually the Reuss bound. On the other hand, the Avalon formation has an optimized value of $\xi = 0.55$, which is only slightly softer than the Hill average. This difference may be related to the microstructural arrangement of organic matter. Figure 3b shows that the best fit effective solid modulus, *K*_*S*_, using ξ is always less than the Hill modulus in all formations studied.

The optimized *p* coefficient in Fig. 2 and Table 4 is equal to 4 or close to it in six of the seven formations, the exception being the Cline shale where *p* = 2, which, if a physically meaningful measure of pore shape, would indicate more equant pores^26^, which may be unrealistic in a shale. Similarly, the more common values of 4 or near 4 seen in the other formations would suggest an effective aspect ratio on the order of 0.2. The optimized *p*’s are consistent with reported values from Hart and Wang^31^ for clay-bearing Berea sandstone. If the values reported here are physically interpretable, as opposed to being purely empirical coefficients, it would suggest that most of the porosity is more equant than crack-like. The total porosity could then be viewed as dominated by large equant pores while flat pores, like those occurring between clay platelets, with aspect ratios that can be less than 0.1, could be incorporated into a “crack density” term; these pores are highly compressible but contribute little to the total porosity^27^. It is possible that there is a tradeoff in the fitting between *p* and *m* with the crack density being accommodated by the *m* values. Irrespective of whether the formation optimized *p*’s can be interpreted directly in physical terms, their stability across formations is encouraging.

The optimized *m* coefficients are higher than values reported by Nafe and Drake^28^ for high porosity shallow marine sediments. From Eq. (5), for a given porosity, the higher *m*, the more compressible the rock frame, suggesting compressible pores. Indeed, the predicted pore space bulk modulus, ${K}_{\phi }$, as shown in the bottom panel of Fig. 2 is usually less than or about equal to the predicted dry frame modulus, *K*_*fr*_. This is an indication of the great importance of varied solid constituent properties. Conversely, one might expect the rock frame compressibility at a given porosity to increase with clay content, due to lower aspect ratio pores, and possibly also with compressible organic content (although this may be accommodated entirely by the calculated effective solid modulus, *K*_*s*_). The data could then possibly be better fit by allowing *m* to be a function of volume fraction of clay^27^ and perhaps that of kerogen by adding additional parameters at the cost of reduced statistical significance of the coefficients. We chose to simplify the optimization by holding *m* constant for each formation, thereby, letting it be representative of the average composition for each depth interval investigated. If more precise predictions are required, additional compositional and pore shape dependence of *m* could be considered. Our initial attempts to add such parameters resulted in reduced statistical significant however.

Nevertheless, the empirical rock physics model fits the measured saturated moduli well (Fig. 3b); the standard error being less than 3 GPa across all formations (see Fig. 2 middle panel and Table 4). Of course, this prediction uses coefficients optimized for each entire formation and could be further improved by zoning the coefficients into subintervals corresponding to distinct geological facies. Evidence of this possibility is seen in Fig. 2 in the Eagle Ford formation when there is a significant drop in percent of calcite and a corresponding gap between the predicted and measured *K*_*ud*_.

The excellent reconstruction of the modulus logs from only three average parameters combined with the sample-by-sample volumetric analysis is indeed encouraging; however, the fluid substitution problem usually does not require prediction of *K*_*ud*_, as it is dynamically measured with velocity logs and density. Rather, the reverse problem involves starting with measured *K*_*ud*_ and predicting how it will change as fluid modulus changes using the optimized parameters. We ask the question: How do our semi-empirical Brown–Korringa model fluid substitution results compare to the Gassmann–Hill approach?

### Comparison to Gassmann–Hill fluid substitution

**Figure 4:**
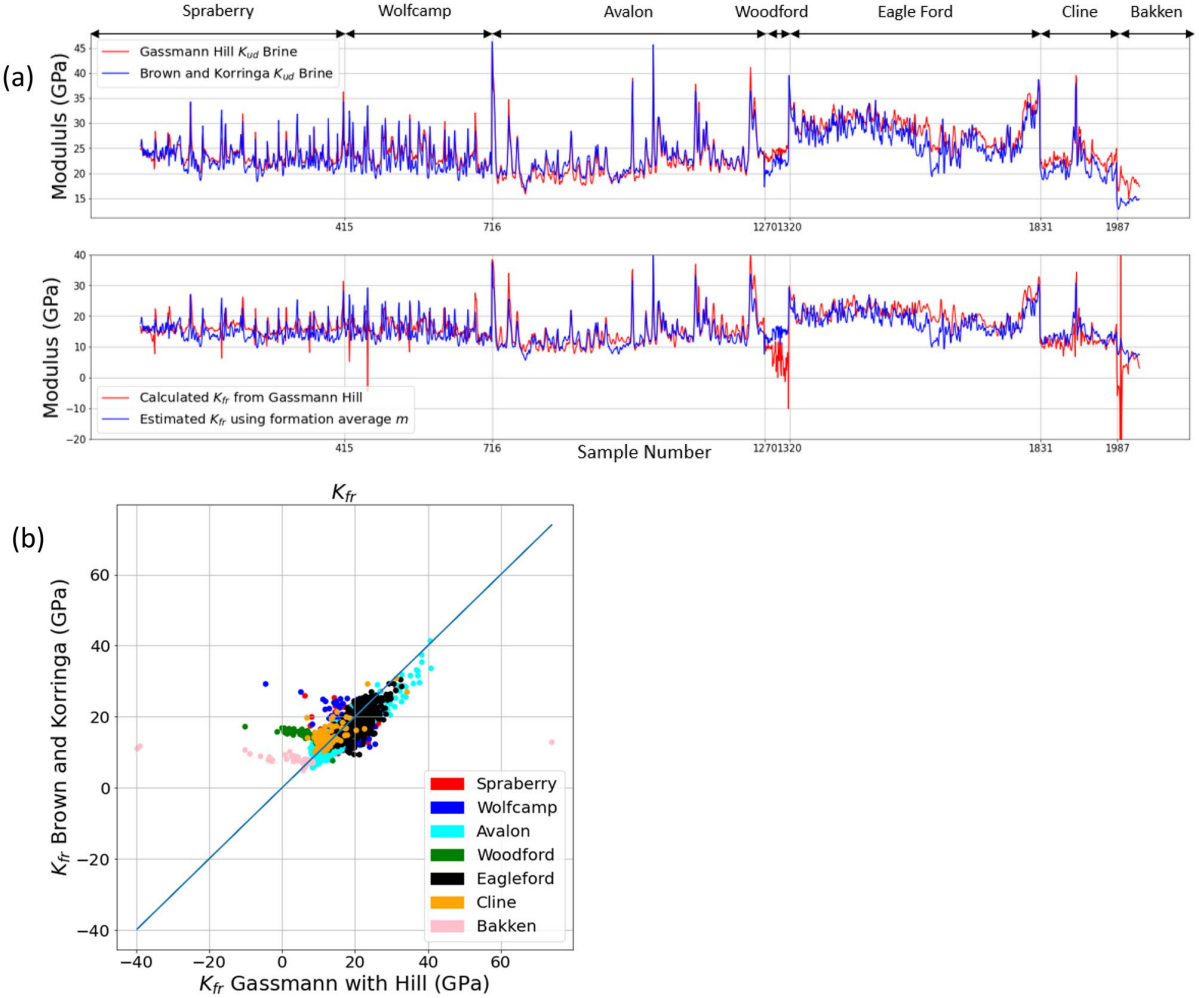
(**a**) Comparison of semi-empirical Brown–Korringa to Gassmann–Hill fluid substitution from in situ saturation to 100% brine saturation. Top panel: predicted saturated bulk modulus at 100% brine saturation for Gassmann–Hill (red curve) and semi-empirical Brown–Korringa (blue curve). Bottom panel: predicted dry-frame bulk modulus, *K*_*fr*_, for Gassmann–Hill (red curve) and semi-empirical Brown–Korringa (blue curve). The Gassmann–Hill dry modulus, *K*_*fr*_, becomes non-physical in some cases with high organic content. (**b**) Crossplot of *K*_*fr*_ predicted by semi-empirical Brown–Korringa model and Gassmann–Hill. Gassmann–Hill yields non-physical or otherwise unreasonable values.

Fluid substitution requires first using the in situ measurements to obtain the original saturated modulus, and then changing the fluid modulus corresponding to the new fluid type, saturation, and/or pressure and temperature conditions. Here we investigate the case of changing the water saturation from the in situ saturation to 100% brine using the Gassmann–Hill approach versus our semi-empirical Brown–Korringa model. In Fig. 4 we show that the Gassmann–Hill dry frame modulus from Eq. (1) goes non-physical in places, particularly in formations with high organic content. Figure 4a highlights that reasonable results for a 100% brine saturated undrained bulk modulus are still obtained even as the dry frame modulus takes on negative values in the second panel. Furthermore, Fig. 4b highlights the value differences between the dry frame modulus predicted from our semi-empirical model and Gassmann-Hill. As the semi-empirical Brown-Korringa frame moduli shown in Fig. 2 are always physical and reasonable, this suggests that using the Hill average results in too high an effective mineral modulus. On the other hand, this error in frame modulus seems to be self-compensating to some extent in the Gassmann-Hill approach, as it results in reasonable 100% brine-saturated moduli. One can show by subtracting Eq. (1) from itself with two different fluid moduli, that the dry frame modulus no longer appears explicitly

5 $$\frac{{K}_{ud2}}{{K}_{S}-{K}_{ud2}}-\frac{{K}_{ud1}}{\phi {(K}_{S}-{K}_{ud1})}=\frac{{K}_{F2}}{\phi {(K}_{S}-{K}_{F2})}-\frac{{K}_{F1}}{{\phi (K}_{S}-{K}_{F1})}$$

where *K*_*ud1*_ and *K*_*F*1_ or the original saturation and fluid modulus, and *K*_*ud2*_ and *K*_*F2*_ are the fluid substituted saturation and modulus. The frame modulus is implicitly contained in the saturated moduli, but fluid substitution can be performed via Eq. (5) without explicitly solving for the frame modulus. Nevertheless, the non-physical frame moduli suggest a weakness in the Gassmann–Hill model which is not apparent in the semi-empirical Brown–Korringa model.

The standard deviation of the discrepancy between Gassmann–Hill and semi-empirical Brown–Korringa saturated moduli at 100% brine saturation is 1.7 GPa for the entire dataset. This is notably less than the in situ discrepancy between measured and predicted saturated moduli In Figs. 2 and 3 and Table 5. The discrepancy is even less when restricting data to less than 5% TOC (1.4 GPa) but increases to 1.9 GPa for TOC greater than 10%. This can be viewed as validating the use of Gassmann-Hill in rocks with low or no organic content when substituting to 100% brine saturation. However, when operating in the reverse direction from fully-brine saturated to gas saturated, the frame modulus dominates, and the discrepancy in frame moduli evident in Fig. 4a, b can be significant.

**Table 5:** The standard deviation of the discrepancy between Gassmann–Hill and semi-empirical Brown–Korringa saturated moduli at 100% brine saturation.

Formation	Standard deviation of ${K}_{sat}$ difference (GPa)	Standard deviation of ${V}_{p}$ difference (km/s)
Spraberry	1.36	0.06
Avalon	0.91	0.05
Bakken	1.12	0.07
Cline	0.86	0.04
Eagle Ford	1.46	0.07
Wolfcamp	1.48	0.07
Woodford	0.74	0.04
Total	1.70	0.09
Less than 5% TOC	1.40	0.07
More than 10% TOC	1.90	0.12

For the entire dataset the standard deviation is only 1.70 GPa for the saturated modulus and 0.09 km/s for the predicted V_p_. However, when the data points are separated by %TOC, the standard deviation for high TOC increases to 1.90 GPa for the saturated modulus and 0.12 km/s for predicted V_p_.

## Conclusions and discussion

We present a novel semi-empirical rock physics model based on the Brown and Korringa^12^ equation for fluid substitution in aggregates with solid constituents having a wide range of bulk moduli, with the idea that it could be better suited for use in shales containing highly compressible solid organic matter. The model also includes (1) the Kachanov et al.^26^ relation between pore compressibility and effective solid bulk modulus which defines a factor *p* which is theoretically related to pore shape, (2) a parameter ξ that relates the effective solid bulk modulus to the Reuss and Voigt bounds, and (3) an empirical relationship between dry frame modulus, effective solid modulus, and porosity used by Nafe and Drake^28^ which contains solid fraction raised by a power *m*.

We test this model in seven different shale formations with a wide range of compositions and fluid properties. We find that optimizing for only three constant empirical coefficients (*p*, ξ and *m*) fits the measured dynamic saturated bulk modulus remarkably well with apparently high statistical significance while yielding physical frame moduli in every case, unlike the Gassmann-Hill approach which goes non-physical at times.

Although the optimized parameters (*p, ξ,* and *m*) are stable and physically reasonable, we view them as simply empirical constants at this stage of model development. The optimized ξ parameter is always greater than 0.5 suggesting that effective solid modulus is always less than the Hill average in organic shales. In most of the formations we find that ξ is close to 1, which corresponds to the Reuss bound, as opposed to the more commonly assumed Hill average. The factor *p* is 3.5 when fit to the entire dataset, which would imply more equant pore shape than we expect in shales and suggests to us that the coefficients *p* and *m* tradeoff to some extent. Future work will involve directly parameterizing the crack density in the rock physics model, which may allow better conceptual interpretation of the optimized parameters, and also optimizing for the effective stress coefficient, *n.*

When substituting to 100% brine saturation, the Gassmann–Hill approach and semi-empirical Brown–Korringa model predict similar saturated moduli, especially for low total organic content. The difference increases with increasing TOC. This may explain the Omovie and Castagna^13^ observation that fluid substituting in situ organic shale measurements to 100% brine saturation using Gassmann-Hill produces results consistent with the Greenberg and Castagna (1992) fully brine-saturated inorganic shale compressional versus shear-wave velocity trend.

In this study, we relied heavily on the assumption that in a given formation at a particular location and over a limited depth interval, we could assume constant empirical constants. In fact, those constants could not have been optimized for in situ, without that assumption over some number of samples. That the data were stably fit well with only three parameters suggests that the assumption is for the most part correct to first order. However, as geological facies vary vertically or laterally, we expect the assumption to break down to some extent. Should significant variability in those parameters occur, it is certainly possible to refine the optimization by zone or geological facies if necessary for practical application.

In conclusion, we have developed a semi-empirical rock physics model that properly handles a mix of solid constituents with very different bulk moduli and have tested it in seven organic shale formations. We find that in every formation, the measured dynamic bulk modulus can be fit well with input compositional volume fractions, constituent properties, and only three empirical coefficients.

## Data Availability

The data used in this paper is available for download as the supplemental information in Omovie and Castagna’s Relationships between Dynamic Elastic Moduli in Shale Reservoirs published in *Energies* at https://www.mdpi.com/1996-1073/13/22/6001 with the direct download link https://www.mdpi.com/1996-1073/13/22/6001/s1?version=1605619595.
